# Effects of a 2-Week 5000 IU versus 1000 IU Vitamin D3 Supplementation on Recovery of Symptoms in Patients with Mild to Moderate Covid-19: A Randomized Clinical Trial

**DOI:** 10.3390/nu13072170

**Published:** 2021-06-24

**Authors:** Shaun Sabico, Mushira A. Enani, Eman Sheshah, Naji J. Aljohani, Dara A. Aldisi, Naif H. Alotaibi, Naemah Alshingetti, Suliman Y. Alomar, Abdullah M. Alnaami, Osama E. Amer, Syed D. Hussain, Nasser M. Al-Daghri

**Affiliations:** 1 Chair for Biomarkers of Chronic Diseases, Biochemistry Department, College of Science, King Saud University, Riyadh 11451, Saudi Arabia; ssabico@ksu.edu.sa (S.S.); najij@hotmail.com (N.J.A.); aalnaami@ksu.edu.sa (A.M.A.); oamer1@ksu.edu.sa (O.E.A.); shussain@ksu.edu.sa (S.D.H.); 2 Infectious Diseases Section, King Fahad Medical City, Riyadh 59046, Saudi Arabia; menani@kfmc.med.sa; 3 Diabetes Care Center, King Salman Bin Abdulaziz Hospital, Riyadh 12769, Saudi Arabia; eman_shesha@hotmail.com; 4 Obesity, Endocrine and Metabolism Center, Department of Medicine, King Fahad Medical City, Riyadh 59046, Saudi Arabia; 5 Department of Community Health Sciences, College of Applied Medical Sciences, King Saud University, Riyadh 11451, Saudi Arabia; daldisi@ksu.edu.sa; 6 Department of Medicine, College of Medicine, King Saud University, Riyadh 12372, Saudi Arabia; dr-alotaibi@hotmail.com; 7 Obstetrics and Gynaecology Department, King Salman Bin Abdulaziz Hospital, Riyadh 11564, Saudi Arabia; alshingetti@yahoo.com; 8 Doping Research Chair, Department of Zoology, College of Science, King Saud University, Riyadh 11495, Saudi Arabia; syalomar@ksu.edu.sa

**Keywords:** COVID-19, vitamin D, clinical trial, saudi, vitamin D insufficiency

## Abstract

Objective: Vitamin D deficiency has been associated with an increased risk of COVID-19 severity. This multi-center randomized clinical trial aims to determine the effects of 5000 IU versus 1000 IU daily oral vitamin D3 supplementation in the recovery of symptoms and other clinical parameters among mild to moderate COVID-19 patients with sub-optimal vitamin D status. Study Design and Setting: A total of 69 reverse transcriptase polymerase chain reaction (RT-PCR) SARS-CoV-2 positive adults who were hospitalized for mild to moderate COVID-19 disease were allocated to receive once daily for 2 weeks either 5000 IU oral vitamin D3 (*n* = 36, 21 males; 15 females) or 1000 IU oral vitamin D3 (standard control) (*n* = 33, 13 males; 20 females). Anthropometrics were measured and blood samples were taken pre- and post-supplementation. Fasting blood glucose, lipids, serum 25(OH)D, and inflammatory markers were measured. COVID-19 symptoms were noted on admission and monitored until full recovery. Results: Vitamin D supplementation for 2 weeks caused a significant increase in serum 25(OH)D levels in the 5000 IU group only (adjusted *p* = 0.003). Within-group comparisons also showed a significant decrease in BMI and IL-6 levels overtime in both groups (*p*-values < 0.05) but was not clinically significant in between-group comparisons. Kaplan–Meier survival analysis revealed that the 5000 IU group had a significantly shorter time to recovery (days) than the 1000 IU group in resolving cough, even after adjusting for age, sex, baseline BMI, and D-dimer (6.2 ± 0.8 versus 9.1 ± 0.8; *p* = 0.039), and ageusia (loss of taste) (11.4 ± 1.0 versus 16.9 ± 1.7; *p* = 0.035). Conclusion: A 5000 IU daily oral vitamin D3 supplementation for 2 weeks reduces the time to recovery for cough and gustatory sensory loss among patients with sub-optimal vitamin D status and mild to moderate COVID-19 symptoms. The use of 5000 IU vitamin D3 as an adjuvant therapy for COVID-19 patients with suboptimal vitamin D status, even for a short duration, is recommended.

## 1. Introduction

The apocalyptic and exponential spread of the coronavirus disease 2019 (COVID-19) has so far claimed almost 4 million human lives globally since it was declared a pandemic in 2020 [1], bringing the entire world to a full stop as it struggled to quickly understand and control the highly contagious severe acute respiratory syndrome coronavirus-2 (SARS-CoV-2), the causative pathogen of COVID-19 [2]. As the months progressed and the strict national lockdowns were eased, it was observed that a large majority of the SARS-CoV-2 carriers were asymptomatic and that the natural course of COVID-19 among infected people eventually led to full recovery, especially if the individual had no pre-existing health conditions [3]. In parallel, advances in COVID-19 management started to increase everything from empirical antivirals and repurposed drugs [4] to the emergency use of potentially efficacious COVID-19 vaccines [5].

Indeed, much has been accomplished by the global medical and academic communities in understanding the etiology and appropriate therapy for COVID-19, especially given the short span of time. In the initial months of the pandemic, a preventive and promising adjuvant therapy was favored given its established role in the prevention of asthmatic exacerbations, viral respiratory infections, pneumonia, and overall mortality in high-risk populations such as the elderly. This well-known supplement is vitamin D [6,7,8,9]. Consequently, accumulating evidence has suggested associations between low levels of vitamin D and the severity of COVID-19 outcome [10,11,12,13]. Among the well-established theories of this association is the biophysical and structural evidence that SARS-CoV-2’s point of cellular entry is the angiotensin converting enzyme 2 (ACE2) receptor protein, which is found in abundance on the surfaces of respiratory cells and is the same point of entry observed in SARS-CoV-1 [14]. Vitamin D heightens the expression of the ACE2 receptor protein, which balances the pathways that are known to be disrupted by coronaviruses, ACE/ACE2 and angiotensin II (ANG)/ANG 1-7 [15,16]. Another interesting theory is that vitamin D is a negative acute phase reactant in most acute and chronic inflammatory conditions [17], which also explains why vitamin D deficiency is common in states that harbor low-grade systemic inflammation such as diabetes, hypertension, heart disease, and aging [18].

Given that both COVID-19 and vitamin D deficiency are global pandemics, and the consistent significant associations between low vitamin D status and many pathologic extra-skeletal conditions including respiratory diseases, clinical trials are thus warranted to provide robust evidence as to whether vitamin D status optimization through supplementation can be preventive and/or therapeutic against coronavirus epidemics. Such empirical investigations are crucial for accurate and up-to-date management as to the true value of vitamin D in the on-going COVID-19 pandemic [19].

As it has already been documented that severe vitamin D deficiency is a predictor of mortality among SA residents [13,20] and that the vitamin D status of confirmed SARS-CoV-2 positive patients are significantly lower than those who tested negative [16], it is the appropriate strategy to move the field forward by conducting intervention trials. To fill this gap, the present randomized, open-label clinical trial aims to determine the beneficial effects of a 2-week, daily 5000 IU versus standard therapy (1000 IU) vitamin D3 supplementation on the recovery times of symptoms among patients having mild to moderate COVID-19 with sub-optimal vitamin D status being treated in tertiary care hospitals in Riyadh, Saudi Arabia.

## 2. Materials and Methods

### 2.1. Study Design and Participants

**Figure 1:**
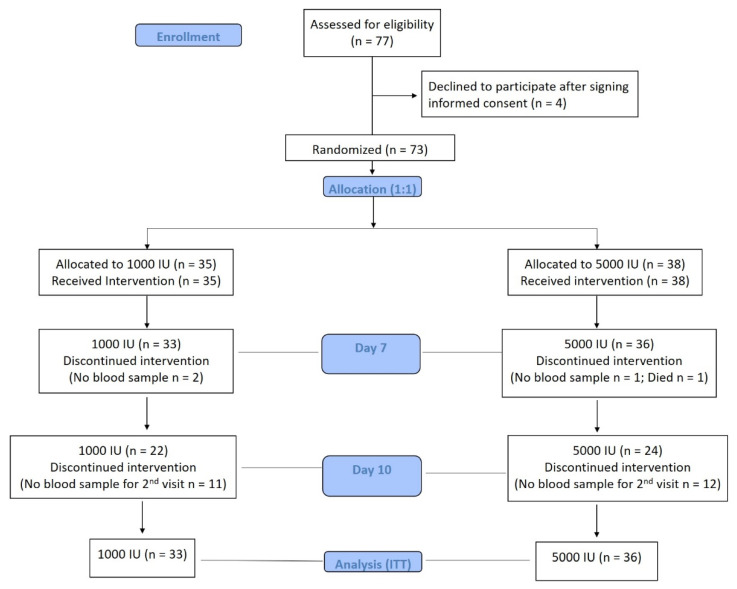
Flowchart of participants.

The present study is a multi-center, randomized clinical trial conducted from 29 July–22 September 2020. In retrospect, the study period coincided with a marked reduction in the daily confirmed COVID-19 cases nationwide (1643 confirmed cases on 29 July 2020 down to 561 cases on 22 September 2020) [21]. Participating centers were all tertiary care hospitals in Riyadh, Kingdom of Saudi Arabia (KSA), and included King Fahad Medical City (KFMC), King Salman Hospital (KSH), and King Saud University Medical City (KSUMC). Male and female adult participants aged 20–75 years old who had an RT-PCR confirmed SARS-CoV-2 positive diagnosis (not more than 3 days prior to inclusion) and were presenting with mild to moderate symptoms and who consented voluntarily (written and verbal) were enrolled in the trial. As per the definition of the Saudi Ministry of Health (MoH) protocol for RT-PCR-confirmed COVID-19 cases, a mild-moderate category meant that the patient required no O2 on presentation, had no evidence of pneumonia but had clinical symptoms (e.g., fever), the management of which was supportive care [22]. Criteria for hospital admission required a confirmed/suspected COVID-19 patient who was symptomatic with evidence of pneumonia, above 65 years, ARDS, the presence of comorbidities and other illnesses that require admission, amongst others (a full list is provided by the MoH hospital admission criteria version 1.1) [23]. Severe COVID-19 cases (those that required intensive care (e.g., respiratory rate ≥30/min, oxygen saturation ≤93%, presence of bilateral lung infiltrates >50% of the lung field)), children and pregnant women, and those whose baseline 25(OH)D were above 75 nmol/L were excluded. Individuals who were SARS-CoV-2 negative and/or SARS-CoV-2 positive but asymptomatic (for home isolation) were also not included. Ethical approval was granted by the Institutional Review Board (IRB) of KFMC, Riyadh, KSA (IRB Log No. 20-282). The study was also registered in the Saudi Clinical Trial Registry (SCTR No. 20061006; Protocol No. H-01-R-012, 7 July 2020) [24]. Figure 1 shows the flowchart of participants. The CONSORT reporting guidelines were used as a checklist for the present randomized trial [25].

### 2.2. Randomization

Patients were allocated (1:1) to receive either standard vitamin D therapy (1000 IU (control)) or 5000 IU vitamin D3 for 14 days. Randomization of the study was done at the KFMC Pharmacy, which also provided the Investigational Drug Service (IDS) clearance and the site for dispensing the supplements. The randomization scheme was computer-generated using four permuted blocks of equal size for the two treatment groups.

### 2.3. Study Protocol

Patients in the 5000 IU group were given Ultra-D^®^ 5000 IU containing 125 µg cholecalciferol (vitamin D3) (Synergy Pharma, Dubai, UAE) while patients in the 1000 IU group were given Vita-D^®^ 1000 IU containing 25 µg cholecalciferol (Synergy Pharma, Dubai, UAE). Both supplements were taken orally daily for 2 weeks. The supplements provided were different in color in both packaging and tablet with unit labels stamped in both the tablet and blister, making blinding of the trial impossible. To monitor compliance, participants were given blisters containing 7 tablets at the baseline visit and were asked to return after one week (Day 7) with any unused tablets for a fresh refill and to monitor symptoms. All participants were advised to continue supplementation until Day 14, even if deisolated/discharged earlier. All participants with pre-existing conditions were advised to continue medications for those pre-existing conditions. Anthropometrics and blood collection were done at baseline (Day 0) and Day 7 or on the discharge day. The monitoring of the primary outcomes (existing symptoms) noted at baseline (Day 0) were followed up on Day 7 or on discharge day and 30 days after discharge and/or the last vitamin dose through a mobile phone call by a data collector who was blind to the treatment received by patients. The primary outcome was the number of days to resolve symptoms. Secondary outcomes include changes in the metabolic profile. Other outcomes such as days to discharge, ICU admission as well as mortality were noted. For the purpose of this study, a recovered case (discharged) was based on the guidelines set by the MoH for symptomatic patients, defined as ‘10 days after onset of symptoms, plus at least 3 days without symptoms (fever and respiratory symptoms) or 3 days without symptoms and one negative RT-PCR test’ [21].

### 2.4. Data Collection

A general questionnaire was administered to all participants, which included demographics, baseline symptoms, medical history, supplements taken as well as baseline anthropometrics. Anthropometrics included height (m), weight (kg), waist (cm), and hip (cm) measurements. Body mass index (BMI) was calculated (kg/m^2^).

All blood sample analyses were sent and carried out in the Biosafety Level 2-facility (BSL-2) with Biological Safety Cabinet Class II (BSC-II), College of American Pathologists (CAP) accredited virology laboratory of KSUMC, Riyadh, SA. Laboratory investigations included complete blood count (including prothrombin time, activated partial thromboplastin time (APTT), international normalized ratio (INR), and bicarbonate), liver profile (bilirubin, bilirubin direct, alkaline phosphatase (ALP), alanine transferase (ALT) and lactate dehydrogenase (LDH)), renal profile (creatinine and urea), inflammatory markers (D-dimer and ferritin), and fasting blood glucose and lipid profile (triglycerides, low-density lipoprotein (LDL-) and high-density lipoprotein (HDL)-cholesterol)), all of which were measured routinely. Interleukin-6 (IL-6) was measured using the Milliplex^®^ MAP Human High Sensitivity T Cell Panel kit (Cat: HSTCMAG) (Millipore Corporation, Billerica, MA, USA) on the FlexMAP 3D System (Luminex Corporation, Austin, TX, USA). The standard curve range for IL-6 is 0.18–750pg/mL, with an inter- and intra-assay coefficient of variation (CVs) of <15% and <10%, respectively. C-reactive protein (CRP) was measured using Maglumi CRP chemiluminescent immunoassays (CLIA) (Shenzhen New Industries Biomedical Engineering Co., Ltd. (SNIBE) Diagnostics, Shenzen, China), with an inter- and intra-assay CVs of <15% and <10%, respectively, and a standard curve range of 0–10,000 µg/mL. Serum 25(OH)D was assessed using the CDC-approved CLIA assays (Maglumi 25OHD, SNIBE Diagnostics, Shenzen, China) as certified by the Vitamin D Standardization-Certification Program (VDSCP) [26], with an assay range of 7.5 nmol/L to 375 nmol/L. Both CRP and 25(OH)D were assessed using a fully automated CLIA analyzer (Maglumi 1000) (SNIBE Diagnostics, Shenzhen, China). Vitamin D deficiency [25(OH)D < 50nmol/L] and vitamin D sufficiency [25(OH)D ≥ 75 nmol/L] were defined based on national and regional recommendations (25, 25). The use of 1000 IU as a control was also based on the standard management of vitamin D deficiency in the GCC region [27,28].

### 2.5. Data Analysis and Sample Size Calculation

Data were entered and analyzed using SPSS version 21.0 (IBM, Chicago, IL, USA). Statistical analysis was performed using intention-to-treat (ITT) analysis, where missing data were managed using the last observation carried forward (LOCF) method. Results were presented as mean ± standard deviation for the continuous normal variables and mean ± standard error (SE) for the continuous non-normal variables. Categorical variables were presented as frequencies (N) and percentages (%). Comparisons between vitamin D doses and other categorical variables were tested using the chi-square test of independence. An independent sample T-test was used to compare clinical variables. Mixed method analysis of covariance (ANCOVA) was used to determine within and between group comparisons overtime, adjusting for baseline covariates age, sex, and BMI. Lastly, Kaplan–Meier survival analysis was done to determine the differences in the recovery time of symptoms, adjusted for age, sex, baseline BMI, and D-dimer. *p*-value < 0.05 was considered significant.

The sample size was taken from published literature [29], reporting a 73% reduction in clinically verified infection (non-SARS-CoV2) among vitamin D deficient patients using vitamin D supplementation. With odds of 0.27 and 80% power, the total required sample size for analysis at a 95% confidence interval (CI) was *n* = 26 (*n* = 13 per arm). A total of 60 cases would thus be recruited to anticipate dropouts (*n* = 30 per arm). A post-hoc power analysis indicated that this study achieved a power of 0.95, with an average difference of 2.9 days between the two doses of vitamin D to resolve cough symptoms, with a standard deviation of 2.8.

## 3. Results

### 3.1. Baseline Characteristics of Participants

A total of 77 participants (*n* = 57 in-patients from KFMC and *n* = 20 outpatients from KSH) were assessed for eligibility (not shown in tables). Table 1 shows the baseline clinical characteristics of the participants overall and after stratification according to vitamin D dose. A total of 69 COVID-19 patients (33 males and 36 females) (mean BMI of 30.7 kg/m^2^ ± 7.8) participated in the present study. The 5000 IU group was significantly younger compared to the 1000 IU group (*p* = 0.03). In contrast, the 1000 IU group had significantly higher BMI than the 5000 IU group (*p* = 0.02). The rest of the baseline anthropometrics and vital signs were not significantly different from one another.

With regard to medical history, hypertension was observed in more than half of all of the participants and was the most common pre-existing condition (55%) followed by type 2 diabetes mellitus (51%), obesity (33%), hyperlipidemia (13%), chronic kidney disease (CKD) (7%), cardiovascular disease (6%), and asthma (4%). No significant differences were found between groups. The rest of the medical history is found in Table 1. The intake of supplements, particularly vitamin C, was noted in 47% of patients. None of the participants claimed to be taking vitamin D supplements prior to COVID-19 diagnosis.

Among the symptoms, fever (77%), dyspnea (71%) muscle pain (59%), and cough (51%) affected more than half of the participants, followed by headache (45%), joint pain (33%), and nausea (25%). Vomiting and sore throat were the least common symptoms (both at 19.2%). No significant differences in the symptoms were seen in both groups. Finally, the clinical conditions of 5 (1%) participants eventually deteriorated and required intensive care. One patient died. The median days to discharge were 7 (CI 5–9). No significant differences were observed in the outcomes of both groups (Table 1). Worthy of note was that vitamin D deficiency was observed in 40 cases (55%), with no difference between the groups (*p* = 0.1), while the rest had vitamin D insufficiency (not shown in table). Other baseline clinical and serologic characteristics of the participants are provided in Supplementary Table S1.

### 3.2. Primary Endpoints

**Figure 2:**
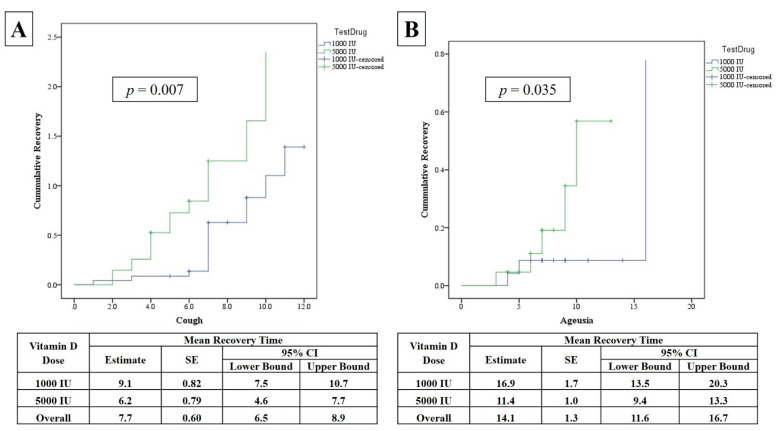
Unadjusted Kaplan–Meier Plot showing the recovery times for cough (**A**) and ageusia (**B**) according to vitamin D dose.

The average days to resolve symptoms in both groups are shown in Table 2. Unadjusted Kaplan–Meier survival analysis was used to determine the differences in recovery times and revealed that the number of days to resolve cough was significantly shorter in the 5000 IU group than the 1000 IU group (6.2 ± 0.8 versus 9.1 ± 0.8; unadjusted *p* = 0.007) (Figure 2A). The same shorter period was observed for ageusia (loss of taste), again in favor of the 5000 IU group (11.4 ± 1.0 versus 16.9 ± 1.7; unadjusted *p* = 0.035) (Figure 2B). None of the other symptom recovery times were significantly different in either groups (Table 2). The significance for cough decreased but persisted even after adjusting for age, sex, baseline BMI, and D-dimer (*p* = 0.039), while the same significance was observed for ageusia (*p* = 0.035) (not mentioned in the figure).

### 3.3. Secondary Endpoints: Clinical Characteristics Overtime

**Figure 3:**
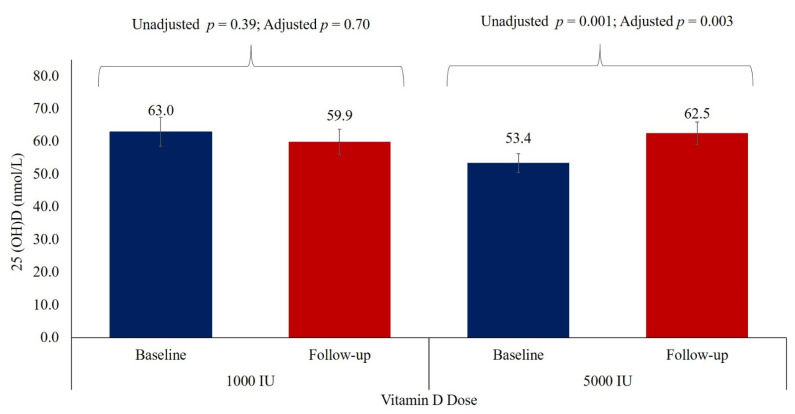
Mean 25(OH) D concentration before and after supplementation.

No adverse events with respect to treatment were reported in either arm. Table 3 shows that within group comparisons, there was a significant decrease in BMI overtime in both the 1000 IU and 5000 IU groups (*p* < 0.05). Furthermore, in both groups, a significant increase was also observed in WBC count, monocyte, ALT, and a significant decrease was seen in levels of IL-6 in (*p* < 0.05) post-intervention. In the 1000 IU group alone, there was a significant increase in hematocrit (*p* = 0.04) and lymphocyte (*p* = 0.03), with a parallel significant decrease in prothrombin time (*p* = 0.05) and ferritin (*p* = 0.004) over time. On the other hand, in the 5000 IU group, there was a significant increase in neutrophil (*p* = 0.03) and urea (*p* < 0.001). Levels of 25(OH)D significantly increased only in the 5000 IU group (*p* = 0.001), and this significance persisted even after the adjustment for covariates (*p* = 0.003) (Figure 3). No significant changes in lipids and glucose were seen in either group post-supplementation. A. Unadjusted between-group comparisons revealed a clinically significant decrease in BMI in favor of the 1000 IU group (*p* = 0.035). This significance was lost after adjustments for baseline BMI, sex, and age (*p* = 0.08). Between-group comparisons revealed no clinically significant differences between the groups with the exception of D-dimer, which was notably higher in the 1000 IU group (Table 3).

## 4. Discussion

To the best of our knowledge, the present study is the first clinical trial for vitamin D and COVID-19 conducted in the Middle East, a region with one of the highest prevalences of vitamin D deficiency in the world, especially in Saudi Arabia (SA) [13,16,20], which consequently, is one of the hardest hit by COVID-19 within the Gulf Cooperation Council (GCC) countries [1]. The goal of the present randomized clinical trial is primarily to determine whether a short-term 5000 IU vitamin D3 supplementation can reduce recovery times of COVID-19 symptoms among mostly in-patients with mild-moderate symptoms. From this trial, it was observed that 5000 IU oral vitamin D3 taken daily for 2 weeks can substantially reduce the days of recovery from cough and ageusia, and this was clinically significant compared to those who took the standard dose for vitamin D deficiency management. It is worth highlighting that the circulating 25(OH)D levels of almost all of the participants at baseline were either in the insufficiency or mild deficiency range, and that 5000 IU vitamin D3 administered for 2 weeks is safe and tolerable, given the acceptable upper safety dose is 4000IU [30]. In a recent case-control study done in KSA, the majority of the 150 hospitalized patients who screened positive for SARS-CoV-2 had severe manifestations of COVID-19 (80% had radiographically confirmed lung infiltrates) and had a much lower vitamin D status (75% had 25(OH)D < 50 nmol) compared to their non-COVID-19 counterparts (*n* = 72), who also experienced severe symptoms but tested negative for SARS-CoV-2 [20]. In comparison, the present participants whose COVID-19 conditions were under the mild to moderate category also had sub-optimal but relatively higher 25(OH)D levels than both groups in the mentioned study. While causality cannot be derived from these observations, the inverse association of 25(OH)D to the severity of COVID-19 outcomes is evident and as such, the possibility of benefitting from vitamin D supplementations needs to be tested.

Preliminary trials on the use of vitamin D supplementation against COVID-19 are limited but accumulating. In a pilot study done in Spain, early high dose vitamin D3 prevented ICU admission among COVID-19 patients in combination with the best available standard care for severe cases [31]. In a case-series of COVID-19 patients who received 50,000 IU daily for 5 days, a marked reduction in recovery time and inflammatory markers were observed compared to those who received 1000 IU [32]. A recent quasi-experimental study also showed that among the frail elderly with COVID-19, those who received boluses of 50,000 IU per month or 80,000–100,000 IU per 2–3 months were associated with less severe and improved survival (OR = 0.08 (0.01: 0.81), *p* = 0.03) [33]. Among the negative trials, a single high dose vitamin D (200,000 IU) given to severe COVID-19 patients (*n* = 114) did not reduce the hospital stay and severity of outcome compared to the placebo group (*n* = 118) (Hazard Ratio 1.12) [34]. The trials mentioned have mostly focused on severe cases and mega-doses of vitamin D compared to the present study, which focused on mild cases and lower daily vitamin D doses. In a large-scale meta-analysis conducted involving almost 11,000 participants in 25 clinical trials on the prevention of acute respiratory infections, the protective effects were the greatest among those vitamin D deficient individuals who received daily or weekly doses as opposed to boluses [9]. The dose used in the present study is somewhat similar to a previous RCT, which demonstrated, albeit during a longer term (12 months), that the supplementation of 4000 IU Vitamin D3 prevented acute respiratory infections by as much as 36% (Relative Risk 0.64, 95 % CI 0.43–0.94) based on a cohort of 140 adults with increased risk of acute respiratory infections (>4 infections/year) [35].

As mentioned previously, the extra-skeletal roles of vitamin D are well-established, not only in respiratory infections but in the regulation of the innate immune system overall. Observations from past coronavirus pandemics such as SARS-CoV-1 demonstrated that coronaviruses inhibit type 1 interferon (IFN) receptors, which inversely affect innate immunity [36]. When unbound, the vitamin D receptor (VDR) deteriorates the beneficial antiviral effects of IFN through the removal of a key transcription factor (STAT1) in IFN signaling. This inverse association between VDR and STAT1 implicates that the unbinding of STAT1 through the increased circulation of biologically active forms of vitamin D (calcitriol) (e.g., supplementation) heightens the type 1 IFN response, consequently improving the innate immune system [37]. Another theory by which elevating the circulating 25(OH)D can enhance coronavirus degradation is the acidification of endolysosomes, cellular organelles in charge of the release of SARS-CoV-2 in the cytosol, thereby stimulating autophagy [38,39]. These mechanisms, together with the ones mentioned previously, may partially explain how vitamin D supplementation can alleviate COVID-19 symptoms, which in the present study includes cough and ageusia. Ageusia is of interest as not much has been published on the role of vitamin D in the reversal of this symptom. Loss of taste and smell however are common in respiratory viral and bacterial infections [40]. Wang and colleagues observed that Toll-like receptor (TLR) and interferon (IFN) pathways were found to be present in taste tissue, and these pathways are activated in response to inflammation (e.g., respiratory infection), which inadvertently interferes normal taste transduction [41]. Vitamin D may restore gustatory function via the suppression of these pathways in the presence of infection, consequently downregulating the inflammatory response [42]. This mechanism is reversed among healthy individuals, where vitamin D may stimulate TLR expression, but in preparation for pathogen exposure [43]. Another explanation can be due to the neuroprotective effects of vitamin D [44], which includes the regulation of the neurotrophins responsible for the development of the gustatory taste system [45].

In the present study, it was apparent that both vitamin D groups had significant reductions in BMI following COVID-19 diagnosis. This observed clinical weight loss can be secondary to the loss of olfactory and gustatory sensations associated with appetite and may have nothing to do with the vitamin D. Unintentional weight loss was observed as one of the collaterals of COVID-19 [46]. Given that most participants in the present study were either overweight or obese, this consequence maybe considered positive for the present cohort, but it also suggests that nutritional therapy may be needed for full recovery of COVID-19 patients following hospital admission and/or isolation [47].

Lastly, while circulating calcium and the parathyroid hormone were not assessed in the present trial, it is important to mention their influence on COVID-19 severity. Calcium in particular plays important roles in virus entry and gene expression [48], with hypocalcemia being commonly observed as a common biochemical abnormality among patients with severe COVID-19 manifestations [49,50], which, in combination with vitamin D deficiency, contributes to a unique osteo-metabolic phenotype [51]. Therefore, vitamin D correction, which controls the entire body’s calcium homeostasis, may further benefit COVID-19 patients with suboptimal 25(OH)D levels by maintaining calcium balance, consequently decreasing risk of COVID-19 severity.

### Strengths and Limitations

The results of the present clinical trial should be interpreted with full consideration of its limitations. Risk of bias is apparent given the study’s open-label design since blinding was impossible. To minimize this, the assessment of symptoms at follow-up were collected over the phone by a blinded data collector. The beneficial effects of 5000 IU vitamin D3 supplementation in this case applies only to mild and moderate COVID-19 cases with sub-optimal vitamin D status (mild deficiency to insufficiency), and whether the same dose and duration will also apply to severe COVID-19 cases with worse vitamin D status needs to be investigated in future clinical trials. The duration of intervention was primarily based on MoH guidelines in terms of deisolation/discharge of COVID-19 cases presenting with mild to moderate symptoms, as it was difficult to monitor the participants physically given the existing COVID-19 restrictions imposed during the study period. While the study had no placebo, the use of 1000 IU is standard and served as a control since it would be deemed unethical not to provide vitamin D supplements if participants were known to have suboptimal vitamin D status. Finally, baseline differences in the parameters were evident despite randomization, as is true for most clinical trials. While age and BMI were used as covariates in all models, these necessary adjustments added stringency to the analysis given its small sample size. Nevertheless, the findings are robust and well powered. The present clinical trial is one of the first interventions globally and the first in the Middle East to use vitamin D as a short-term adjuvant therapy in improving mild to moderate COVID-19 symptoms among patients with sub-optimal vitamin D levels. Prospective cohort studies are needed to determine whether these beneficial effects ultimately extend to prevention of SARS-CoV-2 infection.

## 5. Conclusions

In summary, a 2-week oral supplementation of 5000 IU vitamin D3 was superior to 1000 IU in resolving cough and gustatory sensory loss among COVID-19 patients with sub-optimal vitamin D presenting with mild to moderate symptoms. The present findings add to the growing body of evidence on the beneficial effects of vitamin D supplementation against COVID-19, particularly among those with suboptimal levels.

## Figures and Tables

**Table 1:** Baseline Descriptive Characteristics and Symptoms on Admission.

Parameters	All	1000 IU	5000 IU	*p*-Value
*n*	69	33	36	
Anthropometrics/Vital Signs				
Age	49.8 ± 14.3	53.5 ± 12.3	46.3 ± 15.2	0.03
BMI	30.7 ± 7.8	32.0 ± 6.5	28.2 ± 7.1	0.02
Male/Female	34/35	13/20	21/15	0.12
WHR	0.91 ± 0.11	0.91 ± 0.11	0.90 ± 0.14	0.45
Systolic BP (mmHg)	128.2 ± 17.2	128.3 ± 20.7	128.1 ± 13.4	0.96
Diastolic BP (mmHg)	74.0 ± 13.7	72.8 ± 16.5	75.1 ± 10.6	0.47
Temperature (°C)	37.5 ± 0.9	37.3 ± 0.9	37.7 ± 0.9	0.06
Pulse Rate	93.9 ± 17.2	93.2 ± 17.4	94.5 ± 17.4	0.76
Respiratory Rate	23.9 ± 4.7	24.7 ± 5.0	23.2 ± 4.2	0.19
Medical History (%)				
Hypertension	38 (5)	18 (54)	20 (56)	0.61
T2DM	35 (51)	17 (52)	18 (50)	0.76
Obesity	23 (33)	12 (36)	11 (31)	0.54
Hyperlipidaemia	9 (13)	4 (12)	5 (14)	1.0
CKD	5 (7)	4 (12)	1 (3)	0.19
Cardiovascular Disease	4 (6)	3 (9)	1 (3)	0.34
Asthma	3 (4)	2 (6)	1 (3)	0.60
Rheumatoid	2 (3)	1 (3)	1 (3)	1.0
Thyroid	2 (3)	1 (3)	1 (3)	1.0
Epilepsy	1 (1)	1 (3)	--	1.0
Supplements (%)				
Vitamin C	34 (47)	14 (40)	20 (53)	0.28
Symptoms (%)				
Fever	56 (77)	24 (69)	32 (84)	0.18
Dyspnea	52 (71)	26 (74)	26 (68)	0.58
Fatigue	43 (59)	22 (63)	21 (55)	0.51
Cough	37 (51)	21 (60)	16 (42)	0.28
Headache	33 (45)	13 (37)	20 (53)	0.17
Joint pain	24 (33)	12 (34)	12 (32)	0.85
Nausea	18 (25)	9 (26)	9 (24)	0.31
Diarrhea	16 (22)	8 (23)	8 (21)	0.17
Sore throat	14 (19)	5 (14)	9 (24)	0.17
Vomiting	14 (19)	8 (23)	6 (16)	0.42
Outcomes (N)				
ICU Admission	5	3	2	1.0
Mortality	1	--	1	--
Days to Discharge	7 (5–9)	7 (0–10)	6 (5–8)	0.14

Note: Data presented as N (%) for frequencies and mean ± SD for continuous variables.

**Table 2:** Average Days to Resolve Covid-19 Symptoms according to Vitamin D Dose.

Symptoms	1000 IU	5000 IU	*p*-Value
Fever	9.9 ± 1.7	8.5 ± 0.9	0.97
Dyspnea	11.2 ± 1.6	8.9 ± 1.1	0.24
Fatigue	8.9 ± 0.5	7.7 ± 0.8	0.27
Cough	9.1 ± 0.8	6.2 ± 0.8	0.007
Headache	10.6 ± 0.9	8.7 ± 0.8	0.24
GI symptoms	9.7 ± 1.2	7.6 ± 0.7	0.89
Sore throat	9.5 ± 0.6	12.5 ± 0.7	0.15
Body Aches	9.2 ± 0.9	9.6 ± 0.9	0.68
Chills	17.6 ± 1.2	11.2 ± 1.1	0.14
Anosmia	16.3 ± 1.7	11.2 ± 1.1	0.14
Ageusia	16.9 ± 1.7	11.4 ± 1.0	0.035

Note: Data presented as estimated mean ± SE obtained from Kaplan–Meier survival analysis; *p*-value < 0.05 considered significant.

**Table 3:** Pre and Post Clinical parameters according to Vitamin D supplementation.

Parameters	1000 IU (*n* = 33)			5000 IU (*n* = 36)			Between Group *p*-Value
	Pre-	Post	*p*-Value	Pre-	Post	*p*-Value	
**Anthropometrics**							
BMI (kg/m^2^)	32.0 ± 6.5	31.6 ± 6.0	0.04	28.2 ± 7.1	27.9 ± 5.4	0.049	0.08
WHR	0.91 ± 0.11	0.91 ± 0.1	0.84	0.9 ± 0.14	0.9 ± 0.1	0.65	0.73
**Complete Blood Count**							
Hemoglobin (g/L)	12.7 ± 1.8	13.2 ± 2.2	0.17	13.0 ± 2.8	13.4 ± 2.4	0.03	0.88
Hematocrit (%)	38.5 ± 5.5	40.2 ± 7.2	0.04	40.3 ± 5.7	40.5 ± 6.4	0.66	0.51
RBC count	4.6 ± 0.6	4.8 ± 0.9	0.18	4.8 ± 0.5	4.8 ± 0.7	0.53	0.43
WBC count #	8.5 ± 1.0	9.4 ± 0.9	0.03	6.9 ± 0.4	9.5 ± 0.8	0.001	0.74
Platelet count #	269 ± 29	403 ± 24	<0.001	241 ± 16	380 ± 27	<0.001	0.53
Lymphocyte #	1.0 ± 0.1	1.7 ± 0.2	0.03	2.4 ± 1.1	1.5 ± 0.2	0.95	0.37
Monocyte #	0.5 ± 0.1	0.6 ± 0.1	0.01	0.4 ± 0.0	0.6 ± 0.1	<0.001	0.37
Eosinophil #	0.3 ± 0.1	0.2 ± 0.1	0.85	0.1 ± 0.0	0.1 ± 0.0	0.35	0.30
Neutrophil #	6.2 ± 0.7	6.3 ± 0.5	0.56	5.3 ± 0.5	7.1 ± 0.8	0.03	0.80
Prothrombin Time	13.6 ± 1.6	13.0 ± 1.3	0.05	13.1 ± 1.3	12.9 ± 1.7	0.79	0.76
APTT	32.7 ± 4.8	33.8 ± 7.9	0.85	31.9 ± 4.7	33.8 ± 11.4	0.24	0.74
INR	1.2 ± 0.1	1.1 ± 0.1	0.06	1.1 ± 0.1	1.1 ± 0.1	0.80	0.78
Bicarbonate (mEq/L)	20.8 ± 3.6	22.5 ± 3.1	0.36	21.8 ± 2.7	21.9 ± 6.1	0.51	0.79
**Liver Profile**							
Bilirubin #	7.1 ± 1.2	6.2 ± 0.7	0.65	9.1 ± 1.2	8.8 ± 0.7	0.86	0.06
Bilirubin (direct) #	4.1 ± 0.4	3.9 ± 0.5	0.55	5.3 ± 0.6	4.5 ± 0.3	0.10	0.12
ALP (U/L) #	97.5 ± 16.2	85.9 ± 13.5	0.22	88.5 ± 11.0	106.4 ± 18.6	0.48	0.67
ALT (U/L) #	62.1 ± 17.6	84.7 ± 20.8	0.02	65.3 ± 14.6	114.9 ± 33.5	0.002	0.73
LDH (U/L) #	564 ± 56	484 ± 40	0.32	487 ± 36	410 ± 28	0.16	0.32
**Renal Profile**							
Creatinine (µmol/L)	71.6 ± 16.2	70.9 ± 12.1	0.68	67.0 ± 19.1	66.8 ± 6.3	0.50	0.46
Urea (mg/dl) #	9.1 ± 1.8	8.6 ± 1.7	0.89	5.1 ± 0.5	8.0 ± 1.6	<0.001	0.14
Lipid Profile							
Triglycerides (mmol/L) #	1.5 ± 0.1	2.0 ± 0.2	0.48	1.4 ± 0.1	2.0 ± 0.2	0.36	0.52
Total Cholesterol (mmol/L)	4.0 ± 1.4	4.4 ± 1.4	0.86	4.0 ± 0.9	4.5 ± 1.4	0.97	0.75
HDL-Cholesterol (mmol/L)	1.0 ± 0.2	1.1 ± 0.4	0.39	1.0 ± 0.3	1.1 ± 0.4	0.52	0.48
LDL-Cholesterol (mmol/L)	2.4 ± 1.2	2.4 ± 1.1	0.30	2.3 ± 0.8	2.4 ± 1.1	0.81	0.58
**Inflammatory Markers**							
D-Dimer (µg/mL) #	3.4 ± 2.0	1.9 ± 0.5	0.26	0.6 ± 0.1	1.3 ± 0.6	0.08	0.02
Ferritin (µg/mL) #	784 ± 112	526 ± 76	0.004	733 ± 153	519 ± 96	0.19	0.69
CRP (mg/L) #	47.9 ± 6.8	33.1 ± 7.1	0.10	33.7 ± 5.7	34.2 ± 6.4	0.58	0.25
IL-6 (pg/mL) #	23.9 ± 5.9	19.2 ± 5.6	0.03	18.6 ± 4.6	10.5 ± 2.9	0.01	0.83
Glycemic Profile							
Fasting Glucose (mmol/L) #	10.3 ± 1.1	11.2 ± 1.2	0.38	10.4 ± 1.1	11.4 ± 1.0	0.13	0.91
**Vitamin D**							
25(OH)D (nmol/L) (75–250) #	63.0 ± 2.5	59.9 ± 3.9	0.66	53.4 ± 2.9	62.5 ± 3.4	0.001	0.67

Note: Data presented as mean ± SD for normal variables while mean ± SE for non-normal variables (#); adjusted *p*-values obtained from mixed methods ANCOVA, adjusted for age, sex, and BMI; significant at *p* < 0.05.

## Data Availability

The datasets used and/or analyzed during the current study are available from the corresponding author upon reasonable request.

## Supplementary Materials

The following are available online at https://www.mdpi.com/article/10.3390/nu13072170/s1, Table S1: Baseline Serological Characteristics of Groups.

## Abbreviations

ACE2—Angiotensin converting enzyme; ANCOVA—Analysis of Covariance; BMI—Body mass index; BSC-II—Biological Safety Cabinet Class II; BSL 2—Biosafety Level 2; CAP—College of American Pathologists; CLIA—Chemiluminescent Immunoassay; COVID-19—Coronavirus disease-19; CRP—C-Reactive Protein; GCC—Gulf Cooperation Council; HDL—High density lipoprotein; IFN—Interferon; IL-6—Interleukin 6; IRB—Institutional Review Board; ITT—Intention to treat; KFMC—King Fahad Medical City; KSH—King Salman Hospital; KSUMC—King Saud University Medical City; LDL—Low density lipoprotein; LOCF—Last Observation Carried Forward; RT-PCR—Reverse Transcriptase Polymerase Chain Reaction; SA - Saudi Arabia; SARS-CoV-2—Severe Acute Respiratory Syndrome Coronavirus-2; SD—Standard Deviation; SE – Standard Error; SCTR—Saudi Clinical Trial Registry; SNIBE—Shenzhen New Industries Biomedical Engineering; UAE—United Arab Emirates; VDR—Vitamin D Receptor; VDSCP—Vitamin D Standardization-Certification Program.

## References

[1] Dong E., Du H., Gardner L. (2020). An Interactive Web-Based Dashboard to Track COVID-19 in Real Time. Lancet Infect. Dis..

[2] Coronaviridae Study Group of the International Committee on Taxonomy of Viruses (2020). The species Severe acute respiratory syndrome-related coronavirus: Classifying 2019-nCoV and naming it SARS-CoV-2. Nat. Microbiol..

[3] Oran D.P., Topol E.J. (2020). Prevalence of Asymptomatic SARS-CoV-2 Infection. Ann. Intern. Med..

[4] Sanders J.M., Monogue M.L., Jodlowski T.Z., Cutrell J.B. (2020). Pharmacologic Treatments for Coronavirus Disease 2019 (COVID-19). JAMA.

[5] Lancet T. (2020). COVID-19 Vaccines: No Time for Complacency. Lancet.

[6] Jolliffe D.A., Greenberg L., Hooper R.L., Griffiths C.J., Camargo C.A., Kerley C.P., Jensen M.E., Mauger D., Stelmach I., Urashima M. (2017). Vitamin D supplementation to prevent asthma exacerbations: A systematic review and meta-analysis of individual participant data. Lancet Respir. Med..

[7] Bjelakovic G., Gluud L.L., Nikolova D., Whitfield K., Wetterslev J., Simonetti R.G., Bjelakovic M., Gluud C. (2014). Vitamin D Supplementation for Prevention of Mortality in Adults. Cochrane Database Syst. Rev..

[8] Sarhan T.S., Elrifai A. (2021). Serum Level of Vitamin D as a Predictor for Severity and Outcome of Pneumonia. Clin. Nutr..

[9] Martineau A.R., Jolliffe D.A., Hooper R.L., Greenberg L., Aloia J.F., Bergman P., Dubnov-Raz G., Esposito S., Ganmaa D., Ginde A.A. (2017). Vitamin D supplementation to prevent acute respiratory tract infections: Systematic review and meta-analysis of individual participant data. BMJ.

[10] Ma H., Zhou T., Heianza Y., Qi L. (2021). Habitual Use of Vitamin D Supplements and Risk of Coronavirus Disease 2019 (COVID-19) Infection: A Prospective Study in UK Biobank. Am. J. Clin. Nutr..

[11] Radujkovic A., Hippchen T., Tiwari-Heckler S., Dreher S., Boxberger M., Merle U. (2020). Vitamin D Deficiency and Outcome of COVID-19 Patients. Nutrients.

[12] Mercola J., Grant W.B., Wagner C.L. (2020). Evidence Regarding Vitamin D and Risk of COVID-19 and Its Severity. Nutrients.

[13] Alguwaihes A.M., Al-Sofiani M.E., Megdad M., Albader S.S., Alsari M.H., Alelayan A., Alzahrani S.H., Sabico S., Al-Daghri N.M., Jammah A.A. (2020). Diabetes and Covid-19 among hospitalized patients in Saudi Arabia: A single-centre retrospective study. Cardiovasc. Diabetol..

[14] Wrapp D., Wang N., Corbett K.S., Goldsmith J.A., Hsieh C.-L., Abiona O., Graham B.S., McLellan J.S. (2020). Cryo-EM structure of the 2019-nCoV spike in the prefusion conformation. Science.

[15] Musavi H., Abazari O., Barartabar Z., Kalaki-Jouybari F., Hemmati-Dinarvand M., Esmaeili P., Mahjoub S. (2020). The Benefits of Vitamin D in the COVID-19 Pandemic: Biochemical and Immunological Mechanisms. Arch. Physiol. Biochem..

[16] Al-Daghri N.M., Amer O.E., Alotaibi N.H., Aldisi D.A., Enani M.A., Sheshah E., Aljohani N.J., Alshingetti N., Alomar S.Y., Alfawaz H. (2021). Vitamin D status of Arab Gulf residents screened for SARS-CoV-2 and its association with COVID-19 infection: A multi-centre case-control study. J. Transl. Med..

[17] Waldron J.L., Ashby H.L., Cornes M.P., Bechervaise J., Razavi C., Thomas O.L., Chugh S., Deshpande S., Ford C., Gama R. (2013). Vitamin D: A Negative Acute Phase Reactant. J. Clin. Pathol..

[18] Goncalves de Carvalho C.M., Ribeiro S.M. (2017). Aging, low-grade systemic inflammation and vitamin D: A mini-review. Eur. J. Clin Nutr..

[19] Bassatne A., Basbous M., Chakhtoura M., El Zein O., Rahme M., El-Hajj Fuleihan G. (2021). The link between COVID-19 and Vitamin D (VIVID): A systematic review and meta-analysis. Metabolism.

[20] Alguwaihes A.M., Sabico S., Hasanato R., Al-Sofiani M.E., Megdad M., Albader S.S., Alsari M.H., Alelayan A., Alyusuf E.Y., Alzahrani S.H. (2021). Severe vitamin D deficiency is not related to SARS-CoV-2 infection but may increase mortality risk in hospitalized adults: A retrospective case-control study in an Arab Gulf country. Aging Clin. Exp. Res..

[21] Ministry of Health Saudi Arabia COVID-19 Dashboard. https://covid19.moh.gov.sa.

[22] Saudi MoH Protocol for Patients Suspected of/Confirmed with COVID-19 (Version 2.1 July 2020). https://www.moh.gov.sa/en/Ministry/MediaCenter/Publications/Pages/covid19.aspx.

[23] Saudi MoH Hospital Admission Criteria for COVID-19 Patients (May 2020). https://www.moh.gov.sa/Ministry/MediaCenter/Publications/Documents/admission-criteria.pdf.

[24] Saudi Clinical Trials Registry. https://old.sfda.gov.sa/en/drug/clinical_trials/pages/registered-clinical.aspx.

[25] Schulz K.F., Altman D.G., Moher D. (2010). CONSORT 2010 statement: Updated guidelines for reporting parallel group randomised trials. J. Pharmacol. Pharmacother..

[26] VDSCP Vitamin D Certified Assays Certifications from 2020 (Updated September 2020). https://www.cdc.gov/labstandards/pdf/hs/CDC_Certified_Vitamin_D_Procedures-508.pdf.

[27] Al-Saleh Y., Sulimani R., Sabico S., Raef H., Fouda M., Alshahrani F., Al Shaker M., Al Wahabi B., Sadat-Ali M., Al Rayes H. (2015). 2015 Guidelines for Osteoporosis in Saudi Arabia: Recommendations from the Saudi Osteoporosis Society. Ann. Saudi Med..

[28] Al Saleh Y., Beshyah S.A., Hussein W., Almadani A., Hassoun A., Al Mamari A., Ba-Essa E., Al-Dhafiri E., Hassanein M., Fouda M.A. (2020). Diagnosis and management of vitamin D deficiency in the Gulf Cooperative Council (GCC) countries: An expert consensus summary statement from the GCC vitamin D advisory board. Arch. Osteoporos..

[29] Simpson S., van der Mei I., Stewart N., Blizzard L., Tettey P., Taylor B. (2015). Weekly cholecalciferol supplementation results in significant reductions in infection risk among the vitamin D deficient: Results from the CIPRIS pilot RCT. BMC Nutr..

[30] Rizzoli R. (2021). Vitamin D supplementation: Upper limit for safety revisited?. Aging Clin. Exp. Res..

[31] Entrenas Castillo M., Entrenas Costa L.M., Vaquero Barrios J.M., Alcala Diaz J.F., Lopez Miranda J., Bouillon R., Quesada Gomez J.M. (2020). Effect of calcifediol treatment and best available therapy versus best available therapy on intensive care unit admission and mortality among patients hospitalized for COVID-19: A pilot randomized clinical study. J. Steroid. Biochem. Mol. Biol..

[32] Ohaegbulam K.C., Swalih M., Patel P., Smith M.A., Perrin R. (2020). Vitamin D Supplementation in COVID-19 Patients: A Clinical Case Series. Am. J. Ther..

[33] Annweiler G., Corvaisier M., Gautier J., Dubee V., Legrand E., Sacco G., Annweiler C. (2020). Vitamin D Supplementation Associated to Better Survival in Hospitalized Frail Elderly COVID-19 Patients: The GERIA-COVID Quasi-Experimental Study. Nutrients.

[34] Murai I.H., Fernandes A.L., Sales L.P., Pinto A.J., Goessler K.F., Duran C.S.C., Silva C.B.R., Franco A.S., Macedo M.B., Dalmolin H.H.H. (2021). Effect of a Single High Dose of Vitamin D3 on Hospital Length of Stay in Patients with Moderate to Severe COVID-19: A Randomized Clinical Trial. JAMA.

[35] Bergman P., Norlin A.C., Hansen S., Bjorkhem-Bergman L. (2015). Vitamin D supplementation improves well-being in patients with frequent respiratory tract infections: A post hoc analysis of a randomized, placebo-controlled trial. BMC Res. Notes.

[36] Minakshi R., Padhan K., Rani M., Khan N., Ahmad F., Jameel S. (2009). The SARS Coronavirus 3a protein causes endoplasmic reticulum stress and induces ligand-independent downregulation of the type 1 interferon receptor. PLoS ONE.

[37] Jakovac H. (2020). COVID-19 and vitamin D-Is there a link and an opportunity for intervention?. Am. J. Physiol. Endocrinol. Metab..

[38] Khan N., Chen X., Geiger J.D. (2020). Role of Endolysosomes in Severe Acute Respiratory Syndrome Coronavirus-2 Infection and Coronavirus Disease 2019 Pathogenesis: Implications for Potential Treatments. Front. Pharmacol..

[39] Geiger J.D., Khan N., Murugan M., Boison D. (2020). Possible Role of Adenosine in COVID-19 Pathogenesis and Therapeutic Opportunities. Front. Pharmacol..

[40] Rawson N.E., Huang L. (2009). Symposium overview: Impact of oronasal inflammation on taste and smell. Ann. N. Y. Acad. Sci..

[41] Wang H., Zhou M., Brand J., Huang L. (2009). Inflammation and taste disorders: Mechanisms in taste buds. Ann. N. Y. Acad. Sci..

[42] Do J.E., Kwon S.Y., Park S., Lee E.S. (2008). Effects of vitamin D on expression of Toll-like receptors of monocytes from patients with Behcet’s disease. Rheumatology.

[43] Ojaimi S., Skinner N.A., Strauss B.J., Sundararajan V., Woolley I., Visvanathan K. (2013). Vitamin D deficiency impacts on expression of toll-like receptor-2 and cytokine profile: A pilot study. J. Transl. Med..

[44] Xu Y., Baylink D.J., Chen C.S., Reeves M.E., Xiao J., Lacy C., Lau E., Cao H. (2020). The importance of vitamin d metabolism as a potential prophylactic, immunoregulatory and neuroprotective treatment for COVID-19. J. Transl. Med..

[45] Meng L., Jiang X., Ji R. (2015). Role of neurotrophin in the taste system following gustatory nerve injury. Metab. Brain. Dis..

[46] Di Filippo L., De Lorenzo R., D’Amico M., Sofia V., Roveri L., Mele R., Saibene A., Rovere-Querini P., Conte C. (2021). COVID-19 is associated with clinically significant weight loss and risk of malnutrition, independent of hospitalisation: A post-hoc analysis of a prospective cohort study. Clin. Nutr..

[47] Pironi L., Sasdelli A.S., Ravaioli F., Baracco B., Battaiola C., Bocedi G., Brodosi L., Leoni L., Mari G.A., Musio A. (2021). Malnutrition and nutritional therapy in patients with SARS-CoV-2 disease. Clin. Nutr..

[48] Zhou Y., Frey T.K., Yang J.J. (2009). Viral calciomics: Interplays between Ca^2+^ and virus. Cell Calcium.

[49] Di Filippo L., Doga M., Frara S., Giustina A. (2021). Hypocalcemia in COVID-19: Prevalence, clinical significance and therapeutic implications. Rev. Endocr. Metab. Disord..

[50] Martha J.W., Wibowo A., Pranata R. (2021). Hypocalcemia is associated with severe COVID-19: A systematic review and meta-analysis. Diabetes Metab. Syndr..

[51] Di Filippo L., Frara S., Giustina A. (2021). The emerging osteo-metabolic phenotype of COVID-19: Clinical and pathophysiological aspects. Nat. Rev. Endocrinol..

