# Supplementary material for: Effects of a 2-Week 5000 IU versus 1000 IU Vitamin D3 Supplementation on Recovery of Symptoms in Patients with Mild to Moderate Covid-19: A Randomized Clinical Trial
Source: Nutrients. 2021 Jun 24;13(7):2170. doi: 10.3390/nu13072170 (PMC8308273; doi:10.3390/nu13072170)
Supplement: Supplementary file 1 [file nutrients-13-02170-s001.zip › nutrients-1256881-supplementary.pdf]

Supplementary Table S1. Baseline Serological Characteristics of Groups.

**Parameters**	**1000 IU (*n* = 33)**	**5000 IU (*n* = 36)**	***p*-Value**
**Complete Blood Count**			
Hemoglobin (g/L)	12.7 ± 1.8	13.0 ± 2.8	0.94
Hematocrit (%)	38.5 ± 5.5	40.3 ± 5.7	0.56
RBC count	4.6 ± 0.6	4.8 ± 0.5	0.27
WBC count #	8.5 ± 1.0	6.9 ± 0.4	0.24
Platelet count #	269 ± 29	241 ± 16	0.48
Lymphocyte #	1.0 ± 0.1	2.4 ± 1.1	0.17
Monocyte #	0.5 ± 0.1	0.4 ± 0.0	0.67
Eosinophil #	0.3 ± 0.1	0.1 ± 0.0	0.04
Neutrophil #	6.2 ± 0.7	5.3 ± 0.5	0.34
N/L ratio	7.0 ± 1.0	5.8 ± 1.0	0.12
Prothrombin Time	13.6 ± 1.6	13.1 ± 1.3	0.10
APTT	32.7 ± 4.8	31.9 ± 4.7	0.85
INR	1.2 ± 0.1	1.1 ± 0.1	0.10
Bicarbonate (mEq/L)	20.8 ± 3.6	21.8 ± 2.7	0.16
**Liver Profile**			
Bilirubin #	7.1 ± 1.2	9.1 ± 1.2	0.08
Bilirubin (direct) #	4.1 ± 0.4	5.3 ± 0.6	0.03
ALP (U/L) #	97.5 ± 16.2	88.5 ± 11.0	0.29
ALT (U/L) #	62.1 ± 17.6	65.3 ± 14.6	0.51
LDH (U/L) #	564 ± 56	487 ± 36	0.50
**Renal Profile**			
Creatinine (µmol/L)	71.6 ± 16.2	67.0 ± 19.1	0.29
Urea (mg/dL) #	9.1 ± 1.8	5.1 ± 0.5	0.01
**Lipid Profile**			
Triglycerides (mmol/L) #	1.5 ± 0.1	1.4 ± 0.1	0.38
Total Cholesterol (mmol/L)	4.0 ± 1.4	4.0 ± 0.9	0.91
HDL-Cholesterol (mmol/L)	1.0 ± 0.2	1.0 ± 0.3	0.17
LDL-Cholesterol (mmol/L)	2.4 ± 1.2	2.3 ± 0.8	0.61
**Inflammatory Markers**			
D-Dimer (µg/mL) #	3.4 ± 2.0	0.6 ± 0.1	0.02
Ferritin (µg/mL) #	784 ± 112	733 ± 153	0.52
CRP (mg/L) #	47.9 ± 6.8	33.7 ± 5.7	0.15
IL-6 (pg/mL) #	23.9 ± 5.9	18.6 ± 4.6	0.67

**Glycemic Profile**			
Fasting Glucose (mmol/L) #	10.3 ± 1.1	10.4 ± 1.1	0.83
**Vitamin D**			
25(OH)D (nmol/L) (75-250) #	63.0 ± 2.5	53.4 ± 2.9	0.10

Note: Data presented as mean ± SD for normal variables while Mean ± SE for non-normal variables (#); Adjusted p-values obtained from mixed methods ANCOVA, adjusted for age, sex and BMI; N/L, neutrophil/lymphocyte; Significant at $p < 0.05$.
